# Biomolecules Orchestrating Cardiovascular Calcification

**DOI:** 10.3390/biom11101482

**Published:** 2021-10-07

**Authors:** Yin Tintut, Henry M. Honda, Linda L. Demer

**Affiliations:** 1 Department of Medicine, University of California-Los Angeles, Los Angeles, CA 90095, USA; ytintut@mednet.ucla.edu (Y.T.); HHonda@mednet.ucla.edu (H.M.H.); 2 Department of Physiology, University of California-Los Angeles, Los Angeles, CA 90095, USA; 3 Department of Orthopaedic Surgery, University of California-Los Angeles, Los Angeles, CA 90095, USA; 4 Veterans Affairs Greater Los Angeles Healthcare System, Los Angeles, CA 90073, USA; 5 Department of Bioengineering, University of California-Los Angeles, Los Angeles, CA 90095, USA; 6 The David Geffen School of Medicine, University of California-Los Angeles, 10833 Le Conte Ave, Los Angeles, CA 90095, USA

**Keywords:** cardiovascular, calcification, inflammation, lipids, skeletal

## Abstract

Vascular calcification, once considered a degenerative, end-stage, and inevitable condition, is now recognized as a complex process regulated in a manner similar to skeletal bone at the molecular and cellular levels. Since the initial discovery of bone morphogenetic protein in calcified human atherosclerotic lesions, decades of research have now led to the recognition that the regulatory mechanisms and the biomolecules that control cardiovascular calcification overlap with those controlling skeletal mineralization. In this review, we focus on key biomolecules driving the ectopic calcification in the circulation and their regulation by metabolic, hormonal, and inflammatory stimuli. Although calcium deposits in the vessel wall introduce rupture stress at their edges facing applied tensile stress, they simultaneously reduce rupture stress at the orthogonal edges, leaving the net risk of plaque rupture and consequent cardiac events depending on local material strength. A clinically important consequence of the shared mechanisms between the vascular and bone tissues is that therapeutic agents designed to inhibit vascular calcification may adversely affect skeletal mineralization and vice versa. Thus, it is essential to consider both systems when developing therapeutic strategies.

## 1. Introduction

Vascular calcification is an ectopic calcification triggered by chronic inflammatory conditions and/or mineral imbalance. Previously considered a degenerative, end-stage, and inevitable condition, it is now recognized as a complex process regulated at the molecular and cellular levels by a wide range of metabolic and hormonal stimuli. It shares many regulatory factors and processes with bone formation in the embryonic skeleton, including both endochondral and/or intramembranous forms of mineralization. As a consequence, therapeutic agents designed to inhibit vascular calcification may adversely affect skeletal mineralization and vice versa [1]. In this review, we focus on core biomolecules that regulate the process of calcification in the cardiovascular system. However, there are many more biomolecules involved in vascular calcification that we have not discussed in this review, such as sclerostin, klotho, and microRNA.

## 2. Forms of Artery Wall Calcium Deposits

Arterial calcification consists of calcium phosphate deposits in the forms of hydroxyapatite [Ca_10_(PO_4_)_6_(OH)_2_], whitlockite [Ca_18_Mg_2_(HPO_4_)_2_(PO_4_)_12_] [2], octacalcium phosphate [Ca_8_(HPO_4_)_2_(PO_4_)_4_·5H_2_O], and amorphous calcium phosphate [3]. In skeletal bone, hydroxyapatite is the most abundant mineral form, and whitlockite the second most abundant, occupying up to 25% of human bone [4]. Hydroxyapatite is the most stable form around neutral pH, whereas whitlockite is the most stable form at acidic pH (pH: <4.2). In studies addressing the clinical need for the repair of bone defects, it is known that microspheres of calcium phosphate induce matrix mineralization both in vitro and in vivo. Whitlockite/chitosan (scaffolds) are more effective than hydroxyapatite/chitosan in inducing proliferation and osteogenic differentiation in cultures of mesenchymal stem cells, and they are effective in narrowing experimental calvarial defects in rats [5].

## 3. Types of Cardiovascular Calcification

Three main types of vascular calcifications, according to the location in the artery wall, are intimal, valvular, and medial calcifications. Calcium deposition in the intimal layer and/or valves is usually associated with chronic inflammatory conditions, whereas calcification in the elastin/collagen fibers in the medial layer is associated with renal insufficiency. However, the location of calcium deposition is often not exclusive to one layer of the artery wall. For example, chronic hyperglycemia and kidney dysfunction associated with diabetes lead to oxidative stress, inflammation, and endothelial dysfunction [6,7], and calcification often develops in both intimal and medial layers of the artery wall.

### 3.1. Intimal Calcification

Intimal calcification is associated with atherosclerosis, which is often triggered by hyperlipidemia. Under hyperlipidemic conditions, low-density lipoprotein (LDL) accumulates in the subendothelial space, leading to the inflammation and recruitment of monocytes and macrophages [8]. Cytokines produced by macrophages in this process are also pro-osteogenic. Collagen I, a key component of the bone matrix that helps organize the hydroxyapatite crystal nucleation and propagation, is also present in atherosclerotic plaque. In addition to collagen and calcium mineral, calcific atherosclerotic lesions usually include foam cells (macrophages with endocytosed LDL), cholesterol crystals, other collagens, and regulatory biomolecules, including osteopontin (OPN), matrix GLA protein (MGP), and osteocalcin (OCN), as detailed below.

### 3.2. Valve Calcification

Calcific aortic valve disease is a progressive disorder that ultimately causes a vicious cycle of hemodynamic collapse leading to death. However, no medical therapy is currently available—only surgical or interventional replacement. As with intimal calcification, the accumulation of lipids, lipoproteins, and inflammatory cells also contributes to the pathogenesis of calcific aortic stenosis. The mineral deposits are typically located in the fibrosa layers of the valve leaflets, which face the aorta where they experience turbulent, oscillatory flow that may create an environment conducive to lipid deposition [9,10]. The side of the valves facing the ventricle experiences laminar flow and is usually free of lesions. Interestingly, aortic stenosis is more often fibrotic in women and calcific in men [11].

### 3.3. Medial Calcification

Monckeberg’s medial sclerosis is the classical form of medial vascular calcification. Located primarily in the arteries of the extremities [12], medial calcification is prevalent in patients with type 2 diabetes and those with chronic kidney disease. Histological analyses of coronary atherectomy specimens show that lesions from diabetic patients have larger necrotic cores, more healed plaque ruptures, and greater numbers of large sheet-like calcium deposits as well as deposits that are smaller than 15 µm [13]. Hyperphosphatemia from renal insufficiency triggers a cascade of mineral and hormonal imbalances that contribute to ectopic calcification. Medial calcification is also associated with damaged and fractured elastic fibers. Four stages of medial calcification, classified based on histology, include the following: granular calcifications along the internal elastic membrane; larger calcium deposits with intimal hyperplasia; circumferential enlargement of calcium deposits with medial distortion; and osseous metaplasia [12]. Other causes of medial calcification include the generalized arterial calcification of infancy (GACI), idiopathic basal ganglia calcification (IBGC), pseudoxanthoma elasticum (PXE), and arterial calcification due to the deficiency of CD73 (ACDC) [12]. There are complex and fascinating inter-relationships among the molecular mechanisms of GACI, PXE, and ACDC. Preclinical models of diabetic vascular calcification and mechanistic insights distinguishing diabetic and atherosclerotic calcification have been discussed in a review by Stabley and Towler [14].

## 4. Clinical Consequences

By interfering with the mechanical elastance required for the physiological functions of the cardiovascular system, calcium deposition contributes to many types of cardiovascular disease, including myocardial infarction, heart failure, and cardiac valve stenosis and insufficiency.

### 4.1. Myocardial Infarction

Coronary artery disease continues to be the most common cause of death in the United States, accounting for approximately 13% of all deaths [15]. Coronary artery calcification (CAC) is an established marker of plaque burden, and a doubling of CAC increases the risk of myocardial infarction or death from coronary artery disease by 15%–35% [16]. Therefore, the obtaining of a CAC score from CT scans is now recommended to assess risk, since a CAC score of zero indicates a low atherosclerotic cardiovascular disease (ASCVD) risk for the subsequent 10 years in many patient groups [17]. Once diagnosed with atherosclerotic vascular disease or after a myocardial infarction or stroke, patients are usually prescribed as HMG-CoA reductase inhibitors (“statins”), which prevent the synthesis of cholesterol in the liver. Statins are the most commonly prescribed class of drugs in patients over 60 and the third most commonly prescribed drug (following antidepressants and analgesics) in patients between 20 and 59 [18]. Although it is accepted that statins prevent a subsequent event or death from myocardial infarction by slowing the growth of the overall plaque volume, CAC cannot be used to follow treatment response to statins since they increase the rate of progression of CAC [19]. This may be unique to statins given that another class of cholesterol-lowering drugs, PCSK9 inhibitors, has been reported to cause a smaller increase in the rate of CAC progression when added to statins [20]. In patients undergoing placement of a coronary artery stent for an acute myocardial infarction, approximately 6% of the culprit lesions are severely calcified, and 26% are moderately calcified. These degrees of calcification are associated about a 50% increase in stent complications such as thrombosis or restenosis [21].

### 4.2. Stroke

Each year, approximately 800,000 Americans have a stroke [22]. Unlike CAC, extracranial carotid artery calcification does not appear to associate with risk of future stroke. Some studies show greater carotid calcification associates with more stable plaque [23]. The internal carotid artery supplies the brain through an intracranial portion that forms a tortuous and elastic siphon, which probably serves to reduce pulsatility. The calcification of the carotid siphon, which would interfere with the buffering function, is associated with both large-vessel [24] and small-vessel (lacunar) strokes [25]. For patients undergoing intracranial endovascular treatment for an acute ischemic stroke, the presence of medial (vs. intimal) calcification is associated with better outcomes [26].

### 4.3. Aortic and Peripheral Arterial Disease (PAD)

As with CAC, thoracic aortic calcification is associated with increased risk of noncardiovascular morbidity and mortality and thus may be a marker of biologic aging [27]. Two dramatic and often fatal presentations of aortic disease are aortic dissection and ruptured aortic aneurysms. In one study, medial arterial calcification was seen in 22% of patients with acute dissection and 52% of patients with chronic dissection [28]; however, the role of calcification in dissections or aneurysm rupture remains to be determined. The calcification of the aorta can lead to a less compliant aorta, which increases the work of the heart, potentially leading to certain types of congestive heart failure. Finally, a porcelain aorta is an extreme example of aortic calcification where extensive circumferential mineral deposits preclude the cross-clamping of the aorta during cardiac surgery.

PAD, involving branches from the aorta, most commonly affects the lower extremities. Over eight million people in the US have lower-extremity PAD [29]. Few patients die from it; however, it is a powerful predictor of death due to cardiovascular disease or stroke. In contrast to CAC, peripheral arterial calcification involves both medial and intimal calcification. In specimens from subjects over 70 years of age with multiple risk factors for atherosclerotic disease, intimal calcification is much more common in above-the-knee arteries compared with in below-the-knee arteries (15% vs. 1.6%), whereas medial calcification occurs equally in both (2.4% vs. 2.3%) [30]. Surprisingly, in patients with diabetes, medial arterial calcification is a stronger predictor of cardiovascular mortality compared with intimal calcification [31]. A recent meta-analysis showed that limb amputation is associated with below-the-knee medial arterial calcification [32].

### 4.4. Heart Failure

There were over 80,000 deaths from congestive heart failure in 2018, a 47% increase from 2008 [15]. Approximately half of cases of heart failure are accompanied by reduced ejection fraction (HFrEF), with the most common cause being myocardial infarction. However, the other half of heart failure patients have preserved ejection fraction (HFpEF) where failure is associated with a reduced rate of cardiac filling. The mechanisms by which patients develop HFpEF are being investigated. One possibility is the loss of the Windkessel effect, when calcification causes aortic stiffness. The Windkessel effect depends on elastic rebound during diastole, where the effect is similar to the physiology of intraaortic balloon pump function. Aortic rebound also increases diastolic aortic pressure, which is essential for coronary perfusion. Diastolic pressure filling the coronary vascular tree may also be essential for a garden-hose effect that pulls the ventricle open and may enhance diastolic filling. Aortic and arterial stiffnesses appear to increase the pressure requirement for ventricular filling and decrease cardiac reserves with exercise [33]. While multiple other factors can increase vascular stiffness, such as fibrosis and elastin fragmentation, the greatest increase in stiffness results from calcification [34].

### 4.5. Aortic Stenosis

Aortic valve calcification increases with age. Aortic valve sclerosis and calcification are seen in 20% adults over 65 and in 50% of adults over 85 [35]. The presence of aortic valve sclerosis is associated with a 50% increase in cardiovascular death and myocardial infarction [36]. In addition, aortic valve calcification is strongly correlated with the severity of aortic stenosis [37]. Aortic valve replacement, usually for aortic stenosis, is the second most common cardiac surgical procedure (following coronary artery bypass surgery) with nearly 73,000 transcatheter aortic valve replacements (TAVRs) and over 57,000 surgical procedures in 2019 [38]. In addition, the progression of aortic valve calcification is associated with the progression of calcified but not noncalcified plaque burden [39].

## 5. Regulation of Calcium Deposition

**Figure 1:**
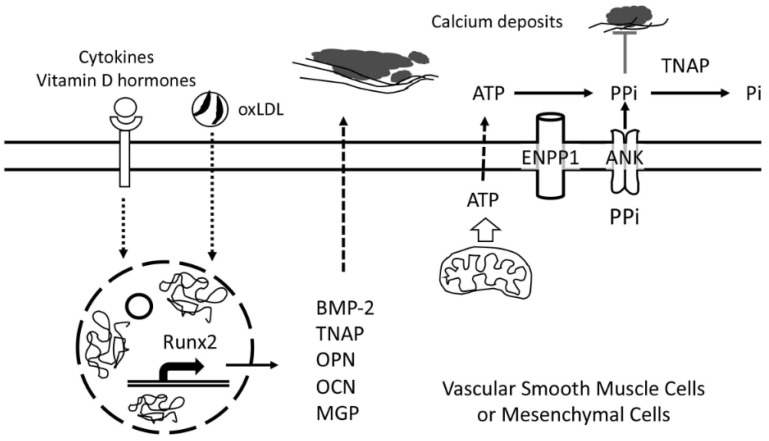
Schematic of some core biomolecules in cardiovascular calcification. Physiologically, extraskeletal cells prevent matrix calcification by producing an inhibitor of calcification, pyrophosphate (PPi). Levels of extracellular PPi is maintained by a plasma membrane transporter, ANK, as well as by a plasma membrane-bound ectoenzyme, ENPP1. In pathological conditions, inflammatory (cytokines, oxLDL), and hormonal (vitamin D hormones) stimuli activate the runt-related transcription factor 2 (Runx-2)-mediated transcription of core biomolecules involved in matrix mineralization, including bone morphogenetic protein (BMP)-2, tissue-nonspecific alkaline phosphatase (TNAP), OPN, OCN, and MGP. The upregulation of TNAP, which is packaged in matrix vesicles and secreted extracellular compartments, contributes to vascular calcification by breaking down PPi.

In general, the calcium mineral appears to form in the extracellular matrix rather than within cells, and the complex process is tightly regulated by a tug-of-war between activators and inhibitors from autocrine, paracrine, endocrine, and exogenous sources. Some of these biomolecules are covered in this review (Table 1). In pathological mineralization of extraskeletal tissues, two dueling biomolecules are as following: an activator, tissue-nonspecific alkaline phosphatase (TNAP), and an inhibitor, inorganic pyrophosphate (PPi). TNAP, which is also considered an active factor in neurodegenerative disease, breaks down PPi into two molecules of phosphate, promoting calcification in two ways, eliminating a potent inhibitor (PPi) and increasing the availability of one of the components of the calcium phosphate mineral (Figure 1). The former is considered of greater significance. This complex feedback circuit may be best evaluated using computational simulation. Extracellular PPi comes from at least two sources; it is generated from extracellular ATP by ectonucleotide pyrophosphatase/phosphodiesterase 1 (Enpp1) and transported to the extracellular space by the membrane transporter, Ank. PPi directly blocks calcium phosphate deposition by interfering with hydroxyapatite crystal nucleation. It may also inhibit further crystal growth by binding to the hydration shell of apatite and hindering inorganic phosphate (Pi) binding [40].

Interestingly, levels of both systemic and extracellular aortic PPi are low in a mouse model of the premature aging disorder, Hutchinson-Gilford progeria syndrome (lamin A mutation) [3], and a key feature of patients with this syndrome is cardiovascular calcification. In this mouse model [3], mitochondrial ATP synthesis is impaired, leading to reduced levels of both intracellular and extracellular ATP. In addition, ectonucleoside triphosphate diphosphohydrolase1 (eNTPD1), which hydrolyzes ATP to inorganic phosphate (Pi), and TNAP are both upregulated. Thus, the low level of PPi in this circumstance is likely a result of low levels of extracellular ATP and the increased expression of TNAP. The plasma PPi levels are also reduced in another mouse model of calcification induced by calcitriol (hypervitaminosis D), but in this case, plasma Pi concentrations are increased [41].

Another inorganic molecule that has the potential to inhibit calcification is magnesium (Mg^2+^), which blocks active growth sites of hydroxyapatite by adsorption at the crystal surface of octacalcium phosphate [42].

## 6. Activator Biomolecules

### 6.1. TNAP

TNAP is a membrane-bound, homodimeric enzyme that catalyzes the hydrolysis of phosphate monoesters in vivo. It has been suggested to dephosphorylate phosphoprotein and endotoxin. There are four isozymes of alkaline phosphatases, and three are tissue-specific, expressed in intestine (ALPI), placenta (ALPP), and germ cells (ALPP2). The fourth enzyme (ALPL) is tissue-nonspecific, expressed in bone, liver, kidney, and brain, among others. Hence, it is designated as TNAP, although the form in the liver has a different glycosylation pattern than those of bone and kidney [43]. TNAP expression is not detectable under physiological conditions but is upregulated in pathological conditions, such as in the aortas of uremic rats and in atherosclerotic lesions [3].

In vivo, the many pathophysiological substrates of TNAP include PPi; adenosine nucleotides [44]; lipopolysaccharide (LPS) [45]; OPN [46]; and pyridoxal phosphate (PLP), which is the major circulating form of vitamin B6 and is required for neurotransmitter synthesis [47]. In vitro substrates include *p*-nitrophenyl phosphate, glucose-6-phosphate, and beta-glycerophosphate [48], which are used for the phosphate source of mineralization in bone and vascular cell cultures.

The role of TNAP in in vivo calcification is to hydrolyze PPi, the potent mineralization inhibitor, allowing the maturation of chondrocytes, and the matrix mineralization of both chondrocytes and vascular smooth muscle cells (VSMCs) [49]. Interestingly, *Tnap^−/−^* mice still have small crystals that fail to grow, suggesting that TNAP may play a role in modulating crystal growth [47]. Recently, Buchet and colleagues found that TNAP in VSMCs also hydrolyzes exogenous ATP in a sequential manner to yield Pi, leading them to posit that TNAP may directly contribute to calcification by generating the phosphate component of the mineral [50]. However, for this to occur, extracellular ATP concentration would need to be in millimolar concentration, which does occur in the tumor microenvironment [51] and conceivably could occur in the atherosclerotic milieu as well.

### 6.2. Bone Morphogenetic Proteins (BMPs)

The first molecular evidence that vascular calcification involves bone regulatory mechanisms was the discovery of the potent osteodifferentiation factor, BMP-2, in human calcific atherosclerotic plaque [52]. BMPs are also powerful morphogens in the transforming growth factor-beta superfamily, and they play crucial roles in the embryonic development of bone, cartilage, and the vasculature [53,54]. BMP gradients guide the spatio-temporal self-organization and differentiation of cells into anatomic patterns in the embryo, a phenomenon reproduced in part in tissue culture. Vascular mesenchymal cells self-organize, undergo osteogenic differentiation and deposit calcium mineral in spatial patterns in culture [55]. The patterns, including spots, stripes, and mazes, are predictable from reaction-diffusion equations based on interactions between BMP-2 and its small-molecule inhibitory morphogen, MGP [56]. Interventions that change the concentrations or activity of MGP change the pattern in a manner predicted by the mathematical model.

Binding of BMP to its ligand activates Smad proteins, which translocate from the cytoplasm into the nucleus for the transcriptional regulation of target genes, including TNAP, runt-related transcription factor 2 (Runx2), OPN, and OCN [54]. BMPs also initiate non-Smad intracellular signaling pathways, including ERK, p38, and JNK [53,54]. BMP activity is modulated by extracellular antagonists, including noggin, chordin, follistatin, ventropin, twisted gastrulation, and MGP [53]. BMP signaling mediates the inflammation-induced calcification of cardiac valve interstitial cells [57]. Aberrant BMP signaling leads to vascular dysfunction and diseases, including vascular calcification [58], aberrant branching patterns in the pulmonary vasculature [59], and pulmonary arterial hypertension [53].

### 6.3. Runx2

Runx2 (also known as Cbfa1, AML3, and Osf2) is a transcription factor expressed in multipotent mesenchymal cells, osteoblast-lineage cells, and chondrocytes. Runx2 is essential for the transcription of genes involve in osteoblastic and osteoclastic differentiation and mineralization, including OPN, OCN, and receptor activator of nuclear factor kappa B ligand (RANKL). The runt-domain region governs most of the DNA-binding function [60], and mutational disruption in this region leads to cleidocranial dysplasia, an autosomal dominant bone disorder [61]. The tamoxifen-induced global deletion of *Runx2* in 4-week-old mice leads to the decreased bone mass and extreme deficiency of osteoblasts, suggesting that Runx2 is required for the differentiation of mesenchymal cells to the osteoblastic lineage [62]. Its expression is regulated by methylation as well as post-transcriptional and post-translational modifications [63]. Its role in the calcification of extraosseous cell/tissues is demonstrated in a mouse model of atherosclerosis, where Runx2 is upregulated and colocalized in calcified atherosclerotic lesions [64], and mice with SMC-specific Runx2 deficiency have normal aortic anatomy but have lower high-fat diet-induced vascular calcification [65].

### 6.4. Calcium-Sensing Receptor (CaSR)

CaSR is a seven-transmembrane-domain receptor, belonging to the family of G-protein-coupled receptors [66]. It is expressed in several organs, including vascular cells [67,68]. It senses extracellular levels of multivalent ions, polyamines, and polypeptides as well as aminoglycoside antibiotics [66,68]. It also activates intracellular signaling pathways for cellular proliferation and differentiation. Nahar-Gohad and colleagues show that aortic SMCs cultured on hydroxyapatite differentiate into osteoblastic cells via the CaSR mediated induction of the BMP-2/Smad-5 signaling pathway [69]. The activation of CaSR induces intracellular signaling, including MEK/ERK and PLC-IP3 for the proliferation and apoptosis of VSMC, respectively [70]. CaSR is also known to mediate oxLDL-induced metalloproteinase induction via the activation of PI3/Akt signaling [71]. Interestingly, in rheumatoid arthritis patients, CaSR expression in circulating monocytes correlated with CAC [72], suggesting the potential pathological role of inflammatory monocytes.

### 6.5. Calcifying Microvesicles

Calcifying cells are well-known to release exosome-like particles known as matrix vesicles. Such vesicles are now recognized as a general mechanism for local, intercellular communication [73]. When vascular smooth muscle cells are exposed to conditions found in chronic kidney disease (CKD), the matrix vesicles (or calcifying vesicles) produce multiple procalcifying signals when taken up by normal vascular smooth muscle cells, thus propagating vascular calcification [74]. These vesicles may also play a role in the induction of vascular calcification by warfarin treatment in patients with cardiac and renal disease. Although one mechanism may be the promotion of BMP-2 activity through the inhibition of MGP, warfarin has also been shown to induce the release of Grp78-loaded calcifying vesicles through endoplasmic reticulum stress [75].

## 7. Inhibitory Biomolecules

In soft extraosseous tissues, under physiological conditions, cells express enzymes and transporters as well as the inhibitory products of these proteins to block calcification, such as fetuin, Enpp1, Ank, osteoprotegerin (OPG), OPN, OCN, MGP, and CD73. Their expression is often found to be increased in calcified arteries, which probably represents a response to the calcification as part of negative feedback regulation.

### 7.1. Fetuin-A

Fetuin-A, also known as a_2_-Heremans-Schmid glycoprotein (AHSG), is a phosphorylated glycoprotein synthesized in the liver and distributed systemically via the bloodstream at concentrations of 0.4 to 0.8 mg/mL in humans and up to several mg/mL in calf sera [76]. Thus, it is absent in avascular tissue such as cartilage. Due to its high affinity to apatite minerals, it is one of the most abundant noncollagenous proteins found in bone [77]. In contrast, its homolog, fetuin-B, lacks the calcification inhibitory function [76]. Based on ex vivo work, in addition to its affinity for calcium phosphate particles, fetuin-A binds to proteins (e.g., TGF-b/BMP) and transports lipids and steroid hormones. Fetuin-A sequesters calciprotein particles from the blood and clears them efficiently via specific macrophage subsets in the liver and kidney [76]. In vivo, fetuin-A deficiency is associated with pathological calcification and bone abnormalities. In humans, it can manifest as infantile cortical hyperostosis (Caffey disease), which is typically due to mutations in collagen I instead. In the calcification-prone DBA/2 mice, it leads to diffuse ectopic calcification, particularly in the thorax, kidneys, and testes [78]. In the atherosclerosis-prone C57BL/6 strain, fetuin A deficiency impairs skeletal growth [79], but surprisingly little ectopic calcification results [80].

### 7.2. ENPP1/ANK

Ectonucleotide pyrophosphatase/phosphodiesterase 1 (ENPP1) and progressive ankylosis protein (ANK or ANKH in human) are central producers of the potent extracellular inhibitor, PPi. ENPP family members (ENPP1-7) are transmembrane metalloenzymes that catabolize nucleotides [81]. ENPP1, also known as plasma-cell antigen-1, hydrolyzes ATP to generate adenosine monophosphate (AMP) and PPi. It is expressed in a variety of tissues, including bone, cartilage, heart, and fat [81]. ENPP1 expression is lower in calcified carotid endarterectomy specimens than in normal or noncalcified samples [82]. In humans, severe ENPP1 deficiency is linked to the syndrome of the generalized arterial calcification of infancy and periarticular calcification [83]. Enpp1-deficient mice also have extensive medial calcification [84,85,86].

ANK is a transmembrane protein that transports PPi and ATP to the extracellular milieu [87]. In humans, mutations in its gene lead to cranio-metaphyseal dysplasia, overgrowth, and sclerosis of the craniofacial bones as well as abnormal modeling of the metaphyses [88]. A knock-in mouse model expressing a human mutation of ANK (Phe377 deletion) has hypomineralized, and the immature bone matrix yet increased bone turnover [89]. Using growth plate chondrocytes, Wang and colleagues showed a regulatory feedback phenomenon, in which altering ANK activity causes complex changes intracellular and extracellular levels of PPi, involving hydrolysis of PPi to Pi by TNAP [90]. As evidence of the functional similarity between ANK and Enpp1, *Ank^–/–^* mice have extensive mineral deposition in articular cartilage, synovial fluid, and arteries [91,92].

### 7.3. OPG

OPG, a secreted glycoprotein expressed in bone, vascular, and immune cells, regulates bone turnover by acting as a soluble decoy receptor for RANKL. Since RANKL from osteoblasts normally triggers RANK-induced osteoclast differentiation leading to bone resorption, by blocking RANKL, OPG prevents bone resorption [93]. Its expression is regulated by a variety of growth factors and cytokines [94]. Its protective role in the vasculature was not appreciated until *Opg^−/−^* mice were found to develop extensive calcification in the aorta and renal arteries [95]. Unexpectedly, in human subjects, high levels of serum OPG have been positively associated with the severity and progression of coronary artery disease, atherosclerosis, and vascular calcification [94]. To address whether OPG acts as a mediator or inhibitor of calcification, Morony and colleagues used recombinant OPG (Fc-OPG) to treat a mouse model of atherosclerosis and found that treatment reduces the calcified lesion area, supporting the role of OPG as an inhibitor rather than a mediator of calcification [96].

### 7.4. OPN

OPN is a major component of cortical bone [97], a phosphoprotein rich in negatively charged amino acids, allowing it to bind to positively charged calcium in hydroxyapatite [98]. The name derives from its function in bridging osteoclasts to bone resorption sites, but it has many other functions in other contexts and other tissues. OPN limits hydroxyapatite growth and, by allowing attachment of osteoclasts, it promotes hydroxyapatite dissolution [99]. It is produced by other cell types, including VSMC, in response to inflammation. *Opn^−/−^* mice develop medial calcification [100,101,102,103]. Since intact phosphorylated OPN limits the propagation of calcification [99], its increased expression in calcified arteries is also likely due to negative feedback regulation.

### 7.5. OCN

OCN, also known as bone glutamic acid (Gla) protein, is a bone-derived, noncollagenous matrix protein found in most vertebrates [104]. It is a 49 amino acid residue that undergoes vitamin K-dependent gamma-carboxylation at one or more of three Gla residues at the amino acid 17, 21, and 24 positions. The negatively charged Gla residues bind to positively charged calcium with high affinity at the surface of the bone minerals. In human carotid endarterectomy specimens, OCN is found to localize in large, calcified areas [105]. In a rapid aortic calcification model in rabbits, Gadeau and colleagues found that OCN expression was detected at 8–14 days after the injury but not at the 2-day time point [106]. In addition to its established role in the structural scaffold of bone, OCN has endocrine functions, including roles in glucose and energy metabolism [107,108] as well as male fertility [109]. Notably, in vivo studies are limited due to differences between human and mouse OCN, including its number of genes, protein sequence conservation, regulation by vitamin D, and pattern of gamma-carboxylation [110].

### 7.6. MGP

MGP is a potent inhibitor of matrix mineralization that is only 15 kDa in size. Like OCN, MGP is post-translationally modified by gamma-glutamyl carboxylase. It has five glutamic acid residues for gamma carboxylation and three serine residues for phosphorylation. The former modification affects its activity as a mineralization inhibitor, whereas the function of the latter is not clear [110]. In humans, MGP deficiency leads to Keutel syndrome, in which tissues composed of soft cartilage, such as the larynx and ear lobes, become calcified. In mice, MGP deficiency leads to the progressive cartilaginous differentiation and calcification of the entire aorta and its branches [111].

Increased expression of MGP has been reported in calcific aortic lesions in rats [112] and in cultured VSMCs under mineralizing conditions [55], evidently a result of a failed compensatory response. MGP protein expression has been detected in human calcified lesions, and its serum levels are increased in patients with atherosclerosis and diabetes [113]. Multiple mechanisms have been identified for its inhibitory effects. One is that MGP binds and inhibits the osteodifferentiation factor, BMP-2 [114]. Due to its small size, resulting in rapid diffusion, and its induction by BMP-2, it can engage in pattern formation through the morphogenic phenomenon of Turing reaction diffusion [56]. Another proposed mechanism is through alterations in elastin, since the calcification in *Mgp^–/–^* mice first appears in elastic laminae and elastin haploinsufficiency rescues the phenotype [115].

The promoter of the gene for MGP contains a cAMP-response element as well as binding sites for the Ets transcription factor family, nuclear transcription factor Y/cytidine-cytidine-adenosine-adenosine-thymidine (NF-Y/CCAAT)-binding factors, and the vitamin D receptor [111]. Genetic studies suggest that blocking vascular calcification in the *Mgp^−/−^* mice by “vascular-specific” MGP overexpression prevents the low bone mass usually found in these mice. However, this may possibly be due to the MGP overexpression in bone marrow stromal cells, since expression driven by the SM22 promoter may affect those cells as well as VSMCs [116].

### 7.7. Ecto-5′-Nucleotidase (CD73)

CD73 is a 5′-ectonucleotidase, which converts extracellular AMP to adenosine. In a fascinating development, St. Hilaire and colleagues identified mutations in *NT5E*, which encodes CD73, in rare patients with an unusual pattern of severe vascular calcification, limited to the lower extremities [117]. Unexpectedly, the CD73-deficient mouse does not develop vascular mineralization [118,119]. Thus, to investigate the mechanism, Moorhead and colleagues utilized CD73-deficient iPSC-derived human mesenchymal cells. They found that CD73 inhibits calcification by activating the A2a/A2b adenosine receptor and downstream cAMP, which inhibits the Akt/FOXO1 induction of TNAP [120,121].

## 8. Regulators of Biomolecules

### 8.1. Lipids/Lipoproteins

Chronic hyperlipidemia promotes atherosclerosis due to the accumulation of lipoproteins in the subendothelial layer of the artery wall, where they are oxidatively modified by cell metabolites [8]. The resultant modified lipids/lipoproteins then elicit potent inflammatory responses in monocyte/macrophages, which produce cytokines, including TNF-alpha and IL-6. Similar processes appear to occur in some forms of cardiac valve disease, especially calcific aortic valve stenosis, which also has some relation to lipids. A genome-wide association study revealed that high levels of circulating lipoprotein (a) [Lp(a)] are associated with aortic valve stenosis and sclerosis [122,123,124]. However, trials of cholesterol-lowering agents have been unsuccessful in reducing progression.

The same lipids and lipoproteins also directly and indirectly induce osteoblastic differentiation and matrix mineralization of vascular and valve cells. Their direct role has been shown by treating VSMCs and valve interstitial cells with lipid oxidation products [124,125,126] or purified Lp(a) [127]. In bovine VSMCs, oxidatively modified lipoproteins as well as oxidized phospholipids induce TNAP activity and matrix mineralization [125]. In endothelial cells, oxidized low-density lipoproteins (oxLDL) induces BMP-2 expression [128,129]. In human aortic valve interstitial cells, oxLDL upregulates BMP-2 levels and synergizes with lipopolysaccharide to augment osteogenic responses by upregulating TNAP [130]. Lipids and lipoproteins also indirectly affect vascular calcification through induction of inflammatory cytokines, such as TNF-alpha, IL1-beta, and IL-6 from monocyte/macrophages activated by oxylipids [127].

### 8.2. Inflammatory Cytokines

Inflammatory cytokines, such as TNF-alpha, IL-6, and IL-1beta, upregulate activators of mineralization. They induce the expression of TNAP, Runx2, and BMP2 and promoting matrix mineralization in extraosseous cells. These include VSMCs, mesenchymal cells, nucleus pulposus cells [131,132,133,134,135], and valve interstitial cells [136]. The mechanism appears to involve the generation of reactive oxygen species. TNF-alpha upregulates Nox4 [137] and p22phox in SMCs [138], whereas in myofibroblasts, TNF-alpha-induced osteogenic programs are mediated by NADPH oxidases [139]. The role of TNF-alpha in aortic calcification was demonstrated in mice using its neutralizing antibody, infliximab, which was found to downregulate BMP2 expression and aortic calcium accumulation [140]. These inflammatory cytokines also downregulate expression of ANK and ENPP1 [131,141]. Interestingly, TNF-alpha promotes matrix calcification in valve interstitial cells that are induced into a myofibroblastic, but not fibroblastic, state by FGF-2 and TGF-beta [142].

Serum levels of inflammatory markers and CAC are increased in patients with diabetes and chronic kidney disease [6,7] as well as in cigarette smokers [143,144]. Thus, antidiabetic drugs that suppress inflammation also suppress calcification in animal models. For example, evogliptin, a dipeptidyl peptidase-4 inhibitor used in type 2 diabetes mellitus, was shown to suppress inflammatory cytokine gene expression and calcific lesions in mice and to reduce matrix calcification in human valve interstitial cell cultures, suggesting a potential therapeutic agent for calcific aortic valve disease [145]. Newer drugs, such as sodium glucose co-transporter-2 inhibitors, also provide beneficial cardiovascular effects [146], at least in part, through reducing circulating levels of inflammatory cytokines [6]. Naturally occurring compounds, such as circulating uromodulin produced in the kidney and anthraquinone emodin, inhibit the osteo-chondrogenic differentiation of VSMCs and valve interstitial cells, in part through interfering with cytokine-induced calcific signaling [147,148].

### 8.3. Vitamin D Hormones

The term “vitamin D” represents a group of lipid-soluble secosteroids, which, when activated, act as steroid hormones. Since the human body synthesizes vitamin D hormones in the presence of sunlight, it is not a true vitamin in the vast majority of people. Vitamin D_2_ is plant-based, whereas vitamin D_3_ is animal-based, and both forms are used as dietary supplements. Both are absorbed with a similar efficiency and activated by sequential hydroxylation reactions occurring in the liver and kidney by the enzymatic activity of 1-alpha hydroxylase [149], which is, importantly, also found in the artery wall. For decades, high doses of vitamin D hormone have been known to cause vascular calcification in humans [149] as well as in animals [150]. Vitamin D hormones have pleiotropic effects in multiple cell types, including bone cells, endothelial cells, VSMCs, monocytes, skeletal muscle cells, and renal cells. It affects the expression of the aforementioned activator biomolecules, including BMP-2, TNAP, OPN, and OCN. Major storage sites of vitamin D hormones are adipose tissue and skeletal muscle [151]. More comprehensive reviews on vitamin D hormones are provided elsewhere [149,152].

## 9. Outlook on Therapeutic Targets

Since calcium deposits introduce rupture risks in the artery wall, removing and/or reducing calcification has been considered a therapeutic goal. As such, strategies have been developed to: 1) resorb calcium deposits using osteoclasts, the natural bone resorbing cells; or 2) halt the progression of calcification. The challenge is that systemic applications of the agents may also have unwanted off-target effects, such as skeletal bone and/or the immune system.

Several attempts have been undertaken to promote resorption of vascular calcium deposits. Spontaneous resorption is rarely seen, and osteoclastic cells are rarely found in the vasculature, even when they are present, they are functionally impaired. This may be due to inhibitors, such as OPG, OPN, and IL-18, produced by VSMCs [153]. Along these lines, Simpson and colleagues [154] attempted to reverse calcification in animal models by delivering osteoclasts exogenously. Their early work in inducing osteoclastic resorption was successful in vitro and in an in vivo model based on the subdermal co-implantation of elastin and osteoclasts in rats [155]. However, the delivery of osteoclasts in vivo poses a number of challenges, and the resorption was ineffective [154]. Using subdermal implants of elastin in rats, which undergo calcification, they delivered osteoclasts by direct injection or in collagen gels, but the cells did not remain at the site of calcification, rendering the treatment ineffective. In another model using abdominal aortic injury, osteoclasts were encapsulated in microbeads for delivery to the site. Although the cells remained at the target site and remained functional, the cells failed to effect resorption because they stayed within the microbeads.

The inverse relationship between vascular calcification and bone mineral density, known as the calcification paradox, offers challenges to developing medical treatments. For instance, while high doses of bisphosphonates can inhibit vascular calcification, they also inhibit bone calcification, resulting in osteomalacia. Conventional doses of bisphosphonates, used in treatment of osteoporosis, fail to reduce or have inconsistent effects on vascular calcification. In one study of patients with CKD, bisphosphonate treatment had no effect on the progression of vascular calcifications [156]. In patients with pseudoxanthoma elasticum (PXE), resulting from a low level of PPi, etidronate seems to be effective in reducing arterial calcification in clinical trials [157,158]. However, in patients with hypercholesterolemia, etidronate treatment has limited effects on atherosclerotic plaques. Etidronate is moderately effective in reducing plaques in the abdominal aorta, but not in the thoracic aorta [159]. However, the combination of etidronate with atorvastatin reduces abdominal aortic plaques in these cohorts more effectively than atorvastatin [159]. The mechanism is not clear.

Another bone-protective factor, OPG, prevents mineral resorption by inhibiting osteoclast differentiation and activation, but it is associated with greater cardiovascular disease. Consistent with other reports, a study of over 100 patients with CKD showed significantly greater all-cause and cardiovascular mortality in those with higher baseline OPG levels [160]. Since monocyte/macrophages and osteoclasts share common myeloid precursors, attempts have been made to activate RANK signaling in myeloid cells in vivo. Jackson and colleagues engineered a fusion protein of RANK driven from the CD68 promoter [161]. The fused protein contains the cytoplasmic domain of RANK and a domain that binds a chemical inducer of dimerization to initiate downstream RANK signaling and osteoclastic differentiation. In earlier in vitro studies, they expressed this construct in a macrophage cell line (RAW264.7), and it induced tartrate-resistant acid phosphatase (a marker for bone tissue) and mineral-resorbing multinucleated osteoclasts [162]. However, when expressed in mice (CD68trans-iRANK), the induction of osteoclastogenesis was not observed, because CD68 was not expressed at earlier time points in osteoclast precursors from the bone marrow and spleen myeloid cells [161].

Other potential therapeutic targets are inhibitors of the activator biomolecules, such as levamisole, which inhibits TNAP. Levamisole is a potent inhibitor, but it also has TNAP-independent effects in neuronal tissue [163], and it may affect cellular immunity. A reportedly more specific and potent TNAP inhibitor is MLS-0038949, which seems to reduce VSMC mineralization [164], although it is unknown whether it has differential effects on bone vs. liver TNAP. Another TNAP inhibitor, SBI-425, appears to be a potent and highly selective inhibitor in mice [165,166,167], although it may have low penetration in tissues [168].

Other inhibitors act on the BMP-signaling pathway. A recent in vitro study suggests that MGP may serve as a potential therapeutic agent. Parashar and colleagues found that an N-terminal peptide of MGP with phosphorylated serine residues prevents the Pi-induced mineralization of elastin membranes in epithelial cell cultures transfected with a tropoelastin expression vector [169]. The pharmacological inhibition of BMP-2 with the small molecule inhibitor, LDN-193189, or the BMP antagonist, ALK3-Fc, also appears to potently inhibit vascular inflammation and calcification in animal models of atherosclerosis [170].

Since the mortality of coronary atherosclerosis is due to plaque rupture causing the thrombotic occlusion of blood flow to myocardium, a key issue is whether calcium deposits increase or decrease plaque rupture. Theoretical analyses suggest that the shape and the location of calcium deposits with respect to each other to the lipid core and to the fibrous cap may influence the level of rupture stress in the vessel wall [171]. Rupture stresses arise in the vascular tissues and atherosclerotic plaques along the edges of mineral deposits on the faces exposed to external tensile forces. The edges facing orthogonal to external forces have reduced rupture stress. Whether the stress leads to rupture depends on the strength of the surrounding tissue, which is heterogenous in atherosclerotic plaque. Thus, deposits with a greater surface area are likely to pose greater risk because there are more sites where the rupture stress may exceed the tissue strength. For a given location, rupture forces generated by a single large calcification are necessarily greater than those generated by a single small deposit. However, for a given amount of a mineral, one that is more porous or fragmented has a lower density and a greater surface area, which may have greater risk. This is consistent with the preliminary finding that high-density deposits appear more stable than low-density deposits [172]. Similarly, ossified mineral deposits, with their fibrous components, have a greater strength than amorphous mineral. Direct mechanical tests of calcific human atherosclerotic lesions showed that toughness of the plaque segments is substantially greater in locations where the mineral has undergone osteoid metaplasia [173,174].

Paradoxically, certain treatments, such as statins and high-intensity exercise, which reduce cardiovascular events, have been shown to increase the progression of CAC [175,176,177]. The mechanisms by which statins affect bone and mineral formation have been reviewed by Chamani and colleagues [178]. These include the increased expression of factors that mediate bone metabolism, including bone morphogenetic protein-2, transforming growth factor-beta, TNAP, and type I collagen. These may account for the increased progression of CAC seen with statin treatment. Controversy remains as to whether statins change the morphology of calcium deposits and whether such a change may account for the conundrum that statins increase an established risk factor for cardiovascular events but reduce cardiovascular risk. In preclinical studies, statins and progressive exercise regimens have been shown to alter the surface area of aortic calcium deposits, as assessed by ^18^F-NaF PET imaging [179,180].

After the initial discovery of BMP in the calcified artery wall [52], suggesting that vascular calcification could be regulated in a manner similar to that of skeletal bone, decades of research have now led to a deeper understanding of regulatory mechanisms and regulatory biomolecules of cardiovascular calcification. Importantly, given the shared mechanisms between skeletal and vascular calcification, it is essential to consider both tissues and perhaps others, when developing therapeutic strategies.

## Figures and Tables

**Table 1:** Biomolecules.

**Activator biomolecules**	**Details**
Tissue-nonspecific alkaline phosphatase (TNAP)	Membrane-bound, homodimeric enzyme Breaking down the calcification inhibitor, PPi Providing a phosphate source for mineralization when PPi levels are high
Bone morphogenetic protein (BMP)	Potent osteodifferentiation factor Inducing expression of Runx2 and osterix
Runt-related transcription factor 2 (Runx2)	Transcription factor Inducing the expression of osteoblastic differentiation genes (e.g., osteocalcin and osteopontin)
Calcium-sensing receptor (CaSR)	Seven-transmembrane-domain, G-protein-coupled receptor Sensing extracellular levels of multivalent ions, polyamines, polypeptides, and aminoglycoside antibiotics Inducing the BMP-2 signaling pathway
Matrix vesicles or calcifying microvesicles	Matrix vesicles for local, intracellular communication Promoting/propagating calcification
**Inhibitory biomolecules**	**Details**
Fetuin-A	Phosphorylated glycoprotein synthesized in the liver Sequestering and clearing calciprotein particles
Ectonucleotide pyrophosphatase/phosphodiesterase 1 (ENPP1)	Transmembrane metalloenzymes Generating PPi from ATP
Ankylosis (ANK)	Transmembrane protein Transporting PPi and ATP to extracellular milieu
Osteoprotegerin (OPG)	Secreted glycoprotein expressed in bone, vascular, and immune cells Blocking RANKL signaling
Osteopontin (OPN)	Phosphoprotein that binds to positively charged calcium in hydroxyapatite Limiting hydroxyapatite growth, allowing osteoclast attachment and promoteing hydroxyapatite dissolution
Osteocalcin (OCN)	Noncollagenous matrix protein post-translationally modified by gamma-glutamyl carboxylation Binding to calcium with high affinity at the surface of bone mineral
Matrix Gla protein (MGP)	Extracellular matrix protein post-translationally modified by gamma-glutamyl carboxylation Inhibiting BMP signaling by binding to BMP
Ecto-5′-nucleotidase (CD73)	Enzyme that converts adenosine monophosphate (AMP) to adenosine Inhibiting calcification by activating the A2a/A2b adenosine receptor

## Data Availability

Not applicable.

## Abbreviations

^18^F-NaF	fluoride-18 labeled sodium fluoride
ACDC	arterial calcification due to deficiency of cluster of differentiation 73
AHSG	a_2_-Heremans-Schmid glycoprotein
ALK-3-Fc	recombinant activin receptor-like kinase 3
ALPI	intestinal alkaline phosphatase
ALPP	placental alkaline phosphatase
ALPP2	germ cell alkaline phosphatase
AML3	acute myeloid leukemia 3
AMP	adenosine monophosphate
Ank	ankyrin
ASCVD	atherosclerotic cardiovascular disease
ATP	adenosine triphosphate
BMP-2	bone morphogenetic protein
CAC	coronary artery calcification
CaSR	calcium sensing receptor
Cbfa1	core binding factor alpha 1
CD73	cluster of differentiation 73
ENPP1	ectonucleotide pyrophosphatase/phosphodiesterase 1
eNTPD1	ectonucleoside triphosphate diphosphohydrolase 1
ERK	extracellular signal-regulated kinase
Fc-OPG	recombinant osteoprotegerin
FGF-2	fibroblast growth factor 2
FOXO1	forkhead box protein 1
GACI	generalized arterial calcification of infants
HFpEF	heart failure with preserved ejection fraction
HFrEF	heart failure with reduced ejection fraction
HMG-CoA	3-hydroxy-3-methylglutaryl-coenzyme A
IBGC	idiopathic basal ganglia calcification
iPSC	induced pluripotent stem cells
JNK	c-Jun N-terminal kinase
LDL	low-density lipoprotein
Lp(a)	lipoprotein(a)
LPS	lipopolysaccharide
MEK	mitogen-activated protein kinase kinase
MGP	matrix gamma-carboxyglutamate protein
MMP-2	matrix metalloproteinase protein 2
NF-Y/CCAAT	nuclear transcription factor Y/cytidine-cytidine-adenosine-adenosine-thymidine
Nox4	nicotinamide adenine dinucleotide phosphate oxidase 4
NT5E	gene encoding cluster of differentiation 73
OCN	osteocalcin
OPG	osteoprotegerin
OPN	osteopontin
Osf2	osteoblast-specific factor 2
oxLDL	oxidized low-density lipoprotein
P22phox NADPH	transmembrane subunit of nicotinamide adenine dinucleotide phosphate oxidase
PCSK9	proprotein convertase subtilisin/kexin type 9
PET	positron emission tomography
Pi	inorganic phosphate
PI3	phosphoinositide 3-kinase
PLC-IP3	phospholipase C-inositol 1,4,5-trisphosphate
PLP	pyridoxal phosphate
PPi	inorganic pyrophosphate
PXE	pseudoxanthoma elasticum
RANK	receptor activator of nuclear factor kappa-B
RANKL	receptor activator of nuclear factor kappa-B ligand
Runx2	runt-related transcription factor 2
SMC	smooth muscle cells
TAVR	transcatheter aortic valve replacement
TGF-b	transforming growth factor beta
TNAP	tissue-nonspecific alkaline phosphatase
TNF-a	tumor necrosis factor-alpha
VSMC	vascular smooth muscle cells

## References

[1] Opdebeeck B., Neven E., Millan J.L., Pinkerton A.B., D’Haese P.C., Verhulst A. (2020). Pharmacological TNAP inhibition efficiently inhibits arterial media calcification in a warfarin rat model but deserves careful consideration of potential physiological bone formation/mineralization impairment. Bone.

[2] Curtze S.C., Kratz M., Steinert M., Vogt S. (2016). Step down Vascular Calcification Analysis using State-of-the-Art Nanoanalysis Techniques. Sci. Rep..

[3] Villa-Bellosta R., Rivera-Torres J., Osorio F.G., Acin-Perez R., Enriquez J.A., Lopez-Otin C., Andres V. (2013). Defective extracellular pyrophosphate metabolism promotes vascular calcification in a mouse model of Hutchinson-Gilford progeria syndrome that is ameliorated on pyrophosphate treatment. Circulation.

[4] Cheng H., Chabok R., Guan X., Chawla A., Li Y., Khademhosseini A., Jang H.L. (2018). Synergistic interplay between the two major bone minerals, hydroxyapatite and whitlockite nanoparticles, for osteogenic differentiation of mesenchymal stem cells. Acta Biomater..

[5] Zhou D., Qi C., Chen Y.X., Zhu Y.J., Sun T.W., Chen F., Zhang C.Q. (2017). Comparative study of porous hydroxyapatite/chitosan and whitlockite/chitosan scaffolds for bone regeneration in calvarial defects. Int. J. Nanomed..

[6] Ghosh S., Luo D., He W., Chen J., Su X., Huang H. (2020). Diabetes and calcification: The potential role of anti-diabetic drugs on vascular calcification regression. Pharmacol. Res..

[7] Kaminska J., Stopinski M., Mucha K., Jedrzejczak A., Golebiowski M., Niewczas M.A., Paczek L., Foroncewicz B. (2019). IL 6 but not TNF is linked to coronary artery calcification in patients with chronic kidney disease. Cytokine.

[8] Navab M., Fogelman A.M., Berliner J.A., Territo M.C., Demer L.L., Frank J.S., Watson A.D., Edwards P.A., Lusis A.J. (1995). Pathogenesis of atherosclerosis. Am. J. Cardiol..

[9] Butcher J.T., Simmons C.A., Warnock J.N. (2008). Mechanobiology of the aortic heart valve. J. Heart Valve Dis..

[10] Yap C.H., Saikrishnan N., Tamilselvan G., Yoganathan A.P. (2012). Experimental measurement of dynamic fluid shear stress on the aortic surface of the aortic valve leaflet. Biomech. Model. Mechanobiol..

[11] Buttner P., Feistner L., Lurz P., Thiele H., Hutcheson J.D., Schlotter F. (2021). Dissecting Calcific Aortic Valve Disease-The Role, Etiology, and Drivers of Valvular Fibrosis. Front. Cardiovasc. Med..

[12] Lanzer P., Boehm M., Sorribas V., Thiriet M., Janzen J., Zeller T., St Hilaire C., Shanahan C. (2014). Medial vascular calcification revisited: Review and perspectives. Eur. Heart J..

[13] Yahagi K., Kolodgie F.D., Lutter C., Mori H., Romero M.E., Finn A.V., Virmani R. (2017). Pathology of Human Coronary and Carotid Artery Atherosclerosis and Vascular Calcification in Diabetes Mellitus. Arter. Thromb. Vasc. Biol..

[14] Stabley J.N., Towler D.A. (2017). Arterial Calcification in Diabetes Mellitus: Preclinical Models and Translational Implications. Arter. Thromb. Vasc. Biol..

[15] Virani S.S., Alonso A., Aparicio H.J., Benjamin E.J., Bittencourt M.S., Callaway C.W., Carson A.P., Chamberlain A.M., Cheng S., Delling F.N. (2021). Heart Disease and Stroke Statistics-2021 Update: A Report From the American Heart Association. Circulation.

[16] Detrano R., Guerci A.D., Carr J.J., Bild D.E., Burke G., Folsom A.R., Liu K., Shea S., Szklo M., Bluemke D.A. (2008). Coronary calcium as a predictor of coronary events in four racial or ethnic groups. N. Engl. J. Med..

[17] Grundy S.M., Stone N.J., Bailey A.L., Beam C., Birtcher K.K., Blumenthal R.S., Braun L.T., de Ferranti S., Faiella-Tommasino J., Forman D.E. (2019). 2018 AHA/ACC/AACVPR/AAPA/ABC/ACPM/ADA/AGS/APhA/ASPC/NLA/PCNA Guideline on the Management of Blood Cholesterol: A Report of the American College of Cardiology/American Heart Association Task Force on Clinical Practice Guidelines. J. Am. Coll. Cardiol..

[18] Gu Q. (2014). Prescription Cholesterol-Lowering Medication Use in Adults Aged 40 and Over: United States, 2003–2012.

[19] Lee S.E., Chang H.J., Sung J.M., Park H.B., Heo R., Rizvi A., Lin F.Y., Kumar A., Hadamitzky M., Kim Y.J. (2018). Effects of Statins on Coronary Atherosclerotic Plaques: The PARADIGM Study. JACC Cardiovasc. Imaging.

[20] Ikegami Y., Inoue I., Inoue K., Shinoda Y., Iida S., Goto S., Nakano T., Shimada A., Noda M. (2018). The annual rate of coronary artery calcification with combination therapy with a PCSK9 inhibitor and a statin is lower than that with statin monotherapy. NPJ Aging Mech. Dis..

[21] Genereux P., Madhavan M.V., Mintz G.S., Maehara A., Palmerini T., Lasalle L., Xu K., McAndrew T., Kirtane A., Lansky A.J. (2014). Ischemic outcomes after coronary intervention of calcified vessels in acute coronary syndromes. Pooled analysis from the HORIZONS-AMI (Harmonizing Outcomes With Revascularization and Stents in Acute Myocardial Infarction) and ACUITY (Acute Catheterization and Urgent Intervention Triage Strategy) TRIALS. J. Am. Coll. Cardiol..

[22] Kleindorfer D.O., Towfighi A., Chaturvedi S., Cockroft K.M., Gutierrez J., Lombardi-Hill D., Kamel H., Kernan W.N., Kittner S.J., Leira E.C. (2021). 2021 Guideline for the Prevention of Stroke in Patients With Stroke and Transient Ischemic Attack: A Guideline From the American Heart Association/American Stroke Association. Stroke.

[23] Yoon W.J., Crisostomo P., Halandras P., Bechara C.F., Aulivola B. (2019). The Use of the Agatston Calcium Score in Predicting Carotid Plaque Vulnerability. Ann. Vasc. Surg..

[24] Bos D., Portegies M.L., van der Lugt A., Bos M.J., Koudstaal P.J., Hofman A., Krestin G.P., Franco O.H., Vernooij M.W., Ikram M.A. (2014). Intracranial carotid artery atherosclerosis and the risk of stroke in whites: The Rotterdam Study. JAMA Neurol..

[25] Hong N.R., Seo H.S., Lee Y.H., Kim J.H., Seol H.Y., Lee N.J., Suh S.I. (2011). The correlation between carotid siphon calcification and lacunar infarction. Neuroradiology.

[26] Compagne K.C.J., Clephas P.R.D., Majoie C., Roos Y., Berkhemer O.A., van Oostenbrugge R.J., van Zwam W.H., van Es A., Dippel D.W.J., van der Lugt A. (2018). Intracranial Carotid Artery Calcification and Effect of Endovascular Stroke Treatment. Stroke.

[27] Thomas I.C., Thompson C.A., Yang M., Allison M.A., Forbang N.I., Michos E.D., McClelland R.L., Budoff M.J., Criqui M.H. (2018). Thoracic Aorta Calcification and Noncardiovascular Disease-Related Mortality. Arter. Thromb. Vasc. Biol..

[28] de Jong P.A., Hellings W.E., Takx R.A., Isgum I., van Herwaarden J.A., Mali W.P. (2014). Computed tomography of aortic wall calcifications in aortic dissection patients. PLoS ONE.

[29] Gerhard-Herman M.D., Gornik H.L., Barrett C., Barshes N.R., Corriere M.A., Drachman D.E., Fleisher L.A., Fowkes F.G., Hamburg N.M., Kinlay S. (2017). 2016 AHA/ACC Guideline on the Management of Patients With Lower Extremity Peripheral Artery Disease: A Report of the American College of Cardiology/American Heart Association Task Force on Clinical Practice Guidelines. Circulation.

[30] Torii S., Mustapha J.A., Narula J., Mori H., Saab F., Jinnouchi H., Yahagi K., Sakamoto A., Romero M.E., Narula N. (2019). Histopathologic Characterization of Peripheral Arteries in Subjects With Abundant Risk Factors: Correlating Imaging With Pathology. JACC Cardiovasc. Imaging.

[31] Niskanen L., Siitonen O., Suhonen M., Uusitupa M.I. (1994). Medial artery calcification predicts cardiovascular mortality in patients with NIDDM. Diabetes Care.

[32] Losurdo F., Ferraresi R., Ucci A., Zanetti A., Clerici G., Zambon A. (2021). Association of infrapopliteal medial arterial calcification with lower-limb amputations in high-risk patients: A systematic review and meta-analysis. Vasc. Med..

[33] Reddy Y.N.V., Andersen M.J., Obokata M., Koepp K.E., Kane G.C., Melenovsky V., Olson T.P., Borlaug B.A. (2017). Arterial Stiffening With Exercise in Patients With Heart Failure and Preserved Ejection Fraction. J. Am. Coll. Cardiol..

[34] Lyle A.N., Raaz U. (2017). Killing Me Unsoftly: Causes and Mechanisms of Arterial Stiffness. Arter. Thromb. Vasc. Biol..

[35] Stewart B.F., Siscovick D., Lind B.K., Gardin J.M., Gottdiener J.S., Smith V.E., Kitzman D.W., Otto C.M. (1997). Clinical factors associated with calcific aortic valve disease. Cardiovascular Health Study. J. Am. Coll. Cardiol..

[36] Otto C.M., Lind B.K., Kitzman D.W., Gersh B.J., Siscovick D.S. (1999). Association of aortic-valve sclerosis with cardiovascular mortality and morbidity in the elderly. N. Engl. J. Med..

[37] Pawade T., Clavel M.A., Tribouilloy C., Dreyfus J., Mathieu T., Tastet L., Renard C., Gun M., Jenkins W.S.A., Macron L. (2018). Computed Tomography Aortic Valve Calcium Scoring in Patients With Aortic Stenosis. Circ. Cardiovasc. Imaging.

[38] Carroll J.D., Mack M.J., Vemulapalli S., Herrmann H.C., Gleason T.G., Hanzel G., Deeb G.M., Thourani V.H., Cohen D.J., Desai N. (2021). STS-ACC TVT Registry of Transcatheter Aortic Valve Replacement. Ann. Thorac. Surg..

[39] Lee S.E., Sung J.M., Andreini D., Al-Mallah M.H., Budoff M.J., Cademartiri F., Chinnaiyan K., Choi J.H., Chun E.J., Conte E. (2021). Association between Aortic Valve Calcification Progression and Coronary Atherosclerotic Plaque Volume Progression in the PARADIGM Registry. Radiology.

[40] Fleisch H., Russell R.G., Straumann F. (1966). Effect of pyrophosphate on hydroxyapatite and its implications in calcium homeostasis. Nature.

[41] Villa-Bellosta R. (2018). Synthesis of Extracellular Pyrophosphate Increases in Vascular Smooth Muscle Cells During Phosphate-Induced Calcification. Arter. Thromb. Vasc. Biol..

[42] Jang H.L., Lee H.K., Jin K., Ahn H.Y., Lee H.E., Nam K.T. (2015). Phase transformation from hydroxyapatite to the secondary bone mineral, whitlockite. J. Mater. Chem. B.

[43] Nosjean O., Koyama I., Goseki M., Roux B., Komoda T. (1997). Human tissue non-specific alkaline phosphatases: Sugar-moiety-induced enzymic and antigenic modulations and genetic aspects. Biochem. J..

[44] Pettengill M., Robson S., Tresenriter M., Millan J.L., Usheva A., Bingham T., Belderbos M., Bergelson I., Burl S., Kampmann B. (2013). Soluble ecto-5’-nucleotidase (5’-NT), alkaline phosphatase, and adenosine deaminase (ADA1) activities in neonatal blood favor elevated extracellular adenosine. J. Biol. Chem..

[45] Bender B., Baranyi M., Kerekes A., Bodrogi L., Brands R., Uhrin P., Bosze Z. (2015). Recombinant human tissue non-specific alkaline phosphatase successfully counteracts lipopolysaccharide induced sepsis in mice. Physiol. Res..

[46] Narisawa S., Yadav M.C., Millan J.L. (2013). In vivo overexpression of tissue-nonspecific alkaline phosphatase increases skeletal mineralization and affects the phosphorylation status of osteopontin. J. Bone Min. Res..

[47] Millan J.L., Whyte M.P. (2016). Alkaline Phosphatase and Hypophosphatasia. Calcif. Tissue Int..

[48] Say J.C., Ciuffi K., Furriel R.P., Ciancaglini P., Leone F.A. (1991). Alkaline phosphatase from rat osseous plates: Purification and biochemical characterization of a soluble form. Biochim. Biophys. Acta.

[49] Fakhry M., Roszkowska M., Briolay A., Bougault C., Guignandon A., Diaz-Hernandez J.I., Diaz-Hernandez M., Pikula S., Buchet R., Hamade E. (2017). TNAP stimulates vascular smooth muscle cell trans-differentiation into chondrocytes through calcium deposition and BMP-2 activation: Possible implication in atherosclerotic plaque stability. Biochim. Biophys. Acta Mol. Basis Dis..

[50] Buchet R., Tribes C., Rouaix V., Doumeche B., Fiore M., Wu Y., Magne D., Mebarek S. (2021). Hydrolysis of Extracellular ATP by Vascular Smooth Muscle Cells Transdifferentiated into Chondrocytes Generates Pi but Not PPi. Int. J. Mol. Sci..

[51] Feng L.L., Cai Y.Q., Zhu M.C., Xing L.J., Wang X. (2020). The yin and yang functions of extracellular ATP and adenosine in tumor immunity. Cancer Cell Int..

[52] Bostrom K., Watson K.E., Horn S., Wortham C., Herman I.M., Demer L.L. (1993). Bone morphogenetic protein expression in human atherosclerotic lesions. J. Clin. Investig..

[53] Cai J., Pardali E., Sanchez-Duffhues G., ten Dijke P. (2012). BMP signaling in vascular diseases. FEBS Lett..

[54] Wan M., Cao X. (2005). BMP signaling in skeletal development. Biochem. Biophys. Res. Commun..

[55] Tintut Y., Parhami F., Bostrom K., Jackson S.M., Demer L.L. (1998). cAMP stimulates osteoblast-like differentiation of calcifying vascular cells. Potential signaling pathway for vascular calcification. J. Biol. Chem..

[56] Garfinkel A., Tintut Y., Petrasek D., Bostrom K., Demer L.L. (2004). Pattern formation by vascular mesenchymal cells. Proc. Natl. Acad. Sci. USA.

[57] Li X., Lim J., Lu J., Pedego T.M., Demer L., Tintut Y. (2015). Protective Role of Smad6 in Inflammation-Induced Valvular Cell Calcification. J. Cell. Biochem..

[58] Nakagawa Y., Ikeda K., Akakabe Y., Koide M., Uraoka M., Yutaka K.T., Kurimoto-Nakano R., Takahashi T., Matoba S., Yamada H. (2010). Paracrine osteogenic signals via bone morphogenetic protein-2 accelerate the atherosclerotic intimal calcification in vivo. Arter. Thromb. Vasc. Biol..

[59] Yao Y., Nowak S., Yochelis A., Garfinkel A., Bostrom K.I. (2007). Matrix GLA protein, an inhibitory morphogen in pulmonary vascular development. J. Biol. Chem..

[60] Levanon D., Negreanu V., Bernstein Y., Bar-Am I., Avivi L., Groner Y. (1994). AML1, AML2, and AML3, the human members of the runt domain gene-family: cDNA structure, expression, and chromosomal localization. Genomics.

[61] Xu W., Chen Q., Liu C., Chen J., Xiong F., Wu B. (2017). A novel, complex RUNX2 gene mutation causes cleidocranial dysplasia. BMC Med. Genet..

[62] Tosa I., Yamada D., Yasumatsu M., Hinoi E., Ono M., Oohashi T., Kuboki T., Takarada T. (2019). Postnatal Runx2 deletion leads to low bone mass and adipocyte accumulation in mice bone tissues. Biochem. Biophys. Res. Commun..

[63] Gomathi K., Akshaya N., Srinaath N., Moorthi A., Selvamurugan N. (2020). Regulation of Runx2 by post-translational modifications in osteoblast differentiation. Life Sci..

[64] Byon C.H., Sun Y., Chen J., Yuan K., Mao X., Heath J.M., Anderson P.G., Tintut Y., Demer L.L., Wang D. (2011). Runx2-upregulated receptor activator of nuclear factor kappaB ligand in calcifying smooth muscle cells promotes migration and osteoclastic differentiation of macrophages. Arter. Thromb. Vasc. Biol..

[65] Sun Y., Byon C.H., Yuan K., Chen J., Mao X., Heath J.M., Javed A., Zhang K., Anderson P.G., Chen Y. (2012). Smooth muscle cell-specific runx2 deficiency inhibits vascular calcification. Circ. Res..

[66] Gerbino A., Colella M. (2018). The Different Facets of Extracellular Calcium Sensors: Old and New Concepts in Calcium-Sensing Receptor Signalling and Pharmacology. Int. J. Mol. Sci..

[67] Alam M.U., Kirton J.P., Wilkinson F.L., Towers E., Sinha S., Rouhi M., Vizard T.N., Sage A.P., Martin D., Ward D.T. (2009). Calcification is associated with loss of functional calcium-sensing receptor in vascular smooth muscle cells. Cardiovasc. Res..

[68] Sundararaman S.S., van der Vorst E.P.C. (2021). Calcium-Sensing Receptor (CaSR), Its Impact on Inflammation and the Consequences on Cardiovascular Health. Int. J. Mol. Sci..

[69] Nahar-Gohad P., Gohad N., Tsai C.C., Bordia R., Vyavahare N. (2015). Rat aortic smooth muscle cells cultured on hydroxyapatite differentiate into osteoblast-like cells via BMP-2-SMAD-5 pathway. Calcif. Tissue Int..

[70] Molostvov G., Fletcher S., Bland R., Zehnder D. (2008). Extracellular calcium-sensing receptor mediated signalling is involved in human vascular smooth muscle cell proliferation and apoptosis. Cell. Physiol. Biochem..

[71] Li H.X., Kong F.J., Bai S.Z., He W., Xing W.J., Xi Y.H., Li G.W., Guo J., Li H.Z., Wu L.Y. (2012). Involvement of calcium-sensing receptor in oxLDL-induced MMP-2 production in vascular smooth muscle cells via PI3K/Akt pathway. Mol. Cell Biochem..

[72] Paccou J., Boudot C., Renard C., Liabeuf S., Kamel S., Fardellone P., Massy Z., Brazier M., Mentaverri R. (2014). Total calcium-sensing receptor expression in circulating monocytes is increased in rheumatoid arthritis patients with severe coronary artery calcification. Arthritis Res..

[73] Hutcheson J.D., Aikawa E. (2018). Extracellular vesicles in cardiovascular homeostasis and disease. Curr. Opin. Cardiol..

[74] Chen N.X., O’Neill K.D., Moe S.M. (2018). Matrix vesicles induce calcification of recipient vascular smooth muscle cells through multiple signaling pathways. Kidney Int..

[75] Furmanik M., van Gorp R., Whitehead M., Ahmad S., Bordoloi J., Kapustin A., Schurgers L.J., Shanahan C.M. (2021). Endoplasmic Reticulum Stress Mediates Vascular Smooth Muscle Cell Calcification via Increased Release of Grp78 (Glucose-Regulated Protein, 78 kDa)-Loaded Extracellular Vesicles. Arter. Thromb. Vasc. Biol..

[76] Brylka L., Jahnen-Dechent W. (2013). The role of fetuin-A in physiological and pathological mineralization. Calcif. Tissue Int..

[77] Termine J.D. (1988). Non-collagen proteins in bone. Ciba Found. Symp..

[78] Schafer C., Heiss A., Schwarz A., Westenfeld R., Ketteler M., Floege J., Muller-Esterl W., Schinke T., Jahnen-Dechent W. (2003). The serum protein alpha 2-Heremans-Schmid glycoprotein/fetuin-A is a systemically acting inhibitor of ectopic calcification. J. Clin. Investig..

[79] Seto J., Busse B., Gupta H.S., Schafer C., Krauss S., Dunlop J.W., Masic A., Kerschnitzki M., Zaslansky P., Boesecke P. (2012). Accelerated growth plate mineralization and foreshortened proximal limb bones in fetuin-A knockout mice. PLoS ONE.

[80] Babler A., Schmitz C., Buescher A., Herrmann M., Gremse F., Gorgels T., Floege J., Jahnen-Dechent W. (2020). Microvasculopathy and soft tissue calcification in mice are governed by fetuin-A, magnesium and pyrophosphate. PLoS ONE.

[81] Roberts F., Zhu D., Farquharson C., Macrae V.E. (2019). ENPP1 in the Regulation of Mineralization and Beyond. Trends Biochem. Sci..

[82] Nitschke Y., Hartmann S., Torsello G., Horstmann R., Seifarth H., Weissen-Plenz G., Rutsch F. (2011). Expression of NPP1 is regulated during atheromatous plaque calcification. J. Cell. Mol. Med..

[83] Rutsch F., Vaingankar S., Johnson K., Goldfine I., Maddux B., Schauerte P., Kalhoff H., Sano K., Boisvert W.A., Superti-Furga A. (2001). PC-1 nucleoside triphosphate pyrophosphohydrolase deficiency in idiopathic infantile arterial calcification. Am. J. Pathol..

[84] Johnson K., Polewski M., van Etten D., Terkeltaub R. (2005). Chondrogenesis mediated by PPi depletion promotes spontaneous aortic calcification in NPP1-/- mice. Arter. Thromb. Vasc. Biol..

[85] Okawa A., Nakamura I., Goto S., Moriya H., Nakamura Y., Ikegawa S. (1998). Mutation in Npps in a mouse model of ossification of the posterior longitudinal ligament of the spine. Nat. Genet..

[86] Rutsch F., Ruf N., Vaingankar S., Toliat M.R., Suk A., Hohne W., Schauer G., Lehmann M., Roscioli T., Schnabel D. (2003). Mutations in ENPP1 are associated with ‘idiopathic’ infantile arterial calcification. Nat. Genet..

[87] Costello J.C., Rosenthal A.K., Kurup I.V., Masuda I., Medhora M., Ryan L.M. (2011). Parallel regulation of extracellular ATP and inorganic pyrophosphate: Roles of growth factors, transduction modulators, and ANK. Connect. Tissue Res..

[88] Nurnberg P., Thiele H., Chandler D., Hohne W., Cunningham M.L., Ritter H., Leschik G., Uhlmann K., Mischung C., Harrop K. (2001). Heterozygous mutations in ANKH, the human ortholog of the mouse progressive ankylosis gene, result in craniometaphyseal dysplasia. Nat. Genet..

[89] Chen I.P., Wang C.J., Strecker S., Koczon-Jaremko B., Boskey A., Reichenberger E.J. (2009). Introduction of a Phe377del mutation in ANK creates a mouse model for craniometaphyseal dysplasia. J. Bone Miner. Res..

[90] Wang W., Xu J., Du B., Kirsch T. (2005). Role of the progressive ankylosis gene (ank) in cartilage mineralization. Mol. Cell. Biol..

[91] Gurley K.A., Chen H., Guenther C., Nguyen E.T., Rountree R.B., Schoor M., Kingsley D.M. (2006). Mineral formation in joints caused by complete or joint-specific loss of ANK function. J. Bone Miner. Res..

[92] Ho A.M., Johnson M.D., Kingsley D.M. (2000). Role of the mouse ank gene in control of tissue calcification and arthritis. Science.

[93] Udagawa N., Takahashi N., Yasuda H., Mizuno A., Itoh K., Ueno Y., Shinki T., Gillespie M.T., Martin T.J., Higashio K. (2000). Osteoprotegerin produced by osteoblasts is an important regulator in osteoclast development and function. Endocrinology.

[94] Reid P., Holen I. (2009). Pathophysiological roles of osteoprotegerin (OPG). Eur. J. Cell Biol..

[95] Bucay N., Sarosi I., Dunstan C.R., Morony S., Tarpley J., Capparelli C., Scully S., Tan H.L., Xu W., Lacey D.L. (1998). osteoprotegerin-deficient mice develop early onset osteoporosis and arterial calcification. Genes Dev..

[96] Morony S., Tintut Y., Zhang Z., Cattley R.C., Van G., Dwyer D., Stolina M., Kostenuik P.J., Demer L.L. (2008). Osteoprotegerin inhibits vascular calcification without affecting atherosclerosis in ldlr(-/-) mice. Circulation.

[97] Oldberg A., Franzen A., Heinegard D. (1986). Cloning and sequence analysis of rat bone sialoprotein (osteopontin) cDNA reveals an Arg-Gly-Asp cell-binding sequence. Proc. Natl. Acad. Sci. USA.

[98] Chen Y., Bal B.S., Gorski J.P. (1992). Calcium and collagen binding properties of osteopontin, bone sialoprotein, and bone acidic glycoprotein-75 from bone. J. Biol. Chem..

[99] Steitz S.A., Speer M.Y., McKee M.D., Liaw L., Almeida M., Yang H., Giachelli C.M. (2002). Osteopontin inhibits mineral deposition and promotes regression of ectopic calcification. Am. J. Pathol..

[100] Matsui Y., Rittling S.R., Okamoto H., Inobe M., Jia N., Shimizu T., Akino M., Sugawara T., Morimoto J., Kimura C. (2003). Osteopontin deficiency attenuates atherosclerosis in female apolipoprotein E-deficient mice. Arter. Thromb. Vasc. Biol..

[101] Paloian N.J., Leaf E.M., Giachelli C.M. (2016). Osteopontin protects against high phosphate-induced nephrocalcinosis and vascular calcification. Kidney Int..

[102] Shao J.S., Sierra O.L., Cohen R., Mecham R.P., Kovacs A., Wang J., Distelhorst K., Behrmann A., Halstead L.R., Towler D.A. (2011). Vascular calcification and aortic fibrosis: A bifunctional role for osteopontin in diabetic arteriosclerosis. Arter. Thromb. Vasc. Biol..

[103] Speer M.Y., McKee M.D., Guldberg R.E., Liaw L., Yang H.Y., Tung E., Karsenty G., Giachelli C.M. (2002). Inactivation of the osteopontin gene enhances vascular calcification of matrix Gla protein-deficient mice: Evidence for osteopontin as an inducible inhibitor of vascular calcification in vivo. J. Exp. Med..

[104] Schwetz V., Pieber T., Obermayer-Pietsch B. (2012). The endocrine role of the skeleton: Background and clinical evidence. Eur. J. Endocrinol..

[105] Bini A., Mann K.G., Kudryk B.J., Schoen F.J. (1999). Noncollagenous bone matrix proteins, calcification, and thrombosis in carotid artery atherosclerosis. Arter. Thromb. Vasc. Biol..

[106] Gadeau A.P., Chaulet H., Daret D., Kockx M., Daniel-Lamaziere J.M., Desgranges C. (2001). Time course of osteopontin, osteocalcin, and osteonectin accumulation and calcification after acute vessel wall injury. J. Histochem. Cytochem..

[107] Lee N.K., Sowa H., Hinoi E., Ferron M., Ahn J.D., Confavreux C., Dacquin R., Mee P.J., McKee M.D., Jung D.Y. (2007). Endocrine regulation of energy metabolism by the skeleton. Cell.

[108] Shao J., Wang Z., Yang T., Ying H., Zhang Y., Liu S. (2015). Bone Regulates Glucose Metabolism as an Endocrine Organ through Osteocalcin. Int. J. Endocrinol..

[109] Oury F., Sumara G., Sumara O., Ferron M., Chang H., Smith C.E., Hermo L., Suarez S., Roth B.L., Ducy P. (2011). Endocrine regulation of male fertility by the skeleton. Cell.

[110] Wen L., Chen J., Duan L., Li S. (2018). Vitamin Kdependent proteins involved in bone and cardiovascular health (Review). Mol. Med. Rep..

[111] Bjorklund G., Svanberg E., Dadar M., Card D.J., Chirumbolo S., Harrington D.J., Aaseth J. (2020). The Role of Matrix Gla Protein (MGP) in Vascular Calcification. Curr. Med. Chem..

[112] Sweatt A., Sane D.C., Hutson S.M., Wallin R. (2003). Matrix Gla protein (MGP) and bone morphogenetic protein-2 in aortic calcified lesions of aging rats. J. Thromb. Haemost..

[113] Schurgers L.J., Dissel P.E., Spronk H.M., Soute B.A., Dhore C.R., Cleutjens J.P., Vermeer C. (2001). Role of vitamin K and vitamin K-dependent proteins in vascular calcification. Z. Kardiol..

[114] Wallin R., Cain D., Hutson S.M., Sane D.C., Loeser R. (2000). Modulation of the binding of matrix Gla protein (MGP) to bone morphogenetic protein-2 (BMP-2). Thromb. Haemost..

[115] Khavandgar Z., Roman H., Li J., Lee S., Vali H., Brinckmann J., Davis E.C., Murshed M. (2014). Elastin haploinsufficiency impedes the progression of arterial calcification in MGP-deficient mice. J. Bone Miner. Res..

[116] Marulanda J., Gao C., Roman H., Henderson J.E., Murshed M. (2013). Prevention of arterial calcification corrects the low bone mass phenotype in MGP-deficient mice. Bone.

[117] St Hilaire C., Ziegler S.G., Markello T.C., Brusco A., Groden C., Gill F., Carlson-Donohoe H., Lederman R.J., Chen M.Y., Yang D. (2011). NT5E mutations and arterial calcifications. N. Engl. J. Med..

[118] Joolharzadeh P., St Hilaire C. (2019). CD73 (Cluster of Differentiation 73) and the Differences Between Mice and Humans. Arter. Thromb. Vasc. Biol..

[119] Li Q., Price T.P., Sundberg J.P., Uitto J. (2014). Juxta-articular joint-capsule mineralization in CD73 deficient mice: Similarities to patients with NT5E mutations. Cell Cycle.

[120] Jin H., St Hilaire C., Huang Y., Yang D., Dmitrieva N.I., Negro A., Schwartzbeck R., Liu Y., Yu Z., Walts A. (2016). Increased activity of TNAP compensates for reduced adenosine production and promotes ectopic calcification in the genetic disease ACDC. Sci. Signal..

[121] Moorhead W.J., Chu C.C., Cuevas R.A., Callahan J.T., Wong R., Regan C., Boufford C.K., Sur S., Liu M., Gomez D. (2020). Dysregulation of FOXO1 (Forkhead Box O1 Protein) Drives Calcification in Arterial Calcification due to Deficiency of CD73 and Is Present in Peripheral Artery Disease. Arter. Thromb. Vasc. Biol..

[122] Glader C.A., Birgander L.S., Soderberg S., Ildgruben H.P., Saikku P., Waldenstrom A., Dahlen G.H. (2003). Lipoprotein(a), Chlamydia pneumoniae, leptin and tissue plasminogen activator as risk markers for valvular aortic stenosis. Eur. Heart J..

[123] Gotoh T., Kuroda T., Yamasawa M., Nishinaga M., Mitsuhashi T., Seino Y., Nagoh N., Kayaba K., Yamada S., Matsuo H. (1995). Correlation between lipoprotein(a) and aortic valve sclerosis assessed by echocardiography (the JMS Cardiac Echo and Cohort Study). Am. J. Cardiol..

[124] Zheng K.H., Tsimikas S., Pawade T., Kroon J., Jenkins W.S.A., Doris M.K., White A.C., Timmers N., Hjortnaes J., Rogers M.A. (2019). Lipoprotein(a) and Oxidized Phospholipids Promote Valve Calcification in Patients With Aortic Stenosis. J. Am. Coll. Cardiol..

[125] Parhami F., Morrow A.D., Balucan J., Leitinger N., Watson A.D., Tintut Y., Berliner J.A., Demer L.L. (1997). Lipid oxidation products have opposite effects on calcifying vascular cell and bone cell differentiation. A possible explanation for the paradox of arterial calcification in osteoporotic patients. Arter. Thromb. Vasc. Biol..

[126] Ting T.C., Miyazaki-Anzai S., Masuda M., Levi M., Demer L.L., Tintut Y., Miyazaki M. (2011). Increased lipogenesis and stearate accelerate vascular calcification in calcifying vascular cells. J. Biol. Chem..

[127] Yu B., Hafiane A., Thanassoulis G., Ott L., Filwood N., Cerruti M., Gourgas O., Shum-Tim D., Al Kindi H., de Varennes B. (2017). Lipoprotein(a) Induces Human Aortic Valve Interstitial Cell Calcification. JACC Basic Transl. Sci..

[128] Su X., Ao L., Shi Y., Johnson T.R., Fullerton D.A., Meng X. (2011). Oxidized low density lipoprotein induces bone morphogenetic protein-2 in coronary artery endothelial cells via Toll-like receptors 2 and 4. J. Biol. Chem..

[129] Zhang M., Zhou S.H., Li X.P., Shen X.Q., Fang Z.F., Liu Q.M., Qiu S.F., Zhao S.P. (2008). Atorvastatin downregulates BMP-2 expression induced by oxidized low-density lipoprotein in human umbilical vein endothelial cells. Circ. J..

[130] Zeng Q., Song R., Ao L., Xu D., Venardos N., Fullerton D.A., Meng X. (2014). Augmented osteogenic responses in human aortic valve cells exposed to oxLDL and TLR4 agonist: A mechanistic role of Notch1 and NF-kappaB interaction. PLoS ONE.

[131] Krzyzanowska A.K., Frawley R.J., Damle S., Chen T., Otero M., Cunningham M.E. (2021). Activation of nuclear factor-kappa B by TNF promotes nucleus pulposus mineralization through inhibition of ANKH and ENPP1. Sci. Rep..

[132] Fong F., Xian J., Demer L.L., Tintut Y. (2021). Serotonin receptor type 2B activation augments TNF-alpha-induced matrix mineralization in murine valvular interstitial cells. J. Cell Biochem..

[133] Lencel P., Delplace S., Pilet P., Leterme D., Miellot F., Sourice S., Caudrillier A., Hardouin P., Guicheux J., Magne D. (2011). Cell-specific effects of TNF-alpha and IL-1beta on alkaline phosphatase: Implication for syndesmophyte formation and vascular calcification. Lab. Investig..

[134] Ding J., Ghali O., Lencel P., Broux O., Chauveau C., Devedjian J.C., Hardouin P., Magne D. (2009). TNF-alpha and IL-1beta inhibit RUNX2 and collagen expression but increase alkaline phosphatase activity and mineralization in human mesenchymal stem cells. Life Sci..

[135] Tintut Y., Patel J., Parhami F., Demer L.L. (2000). Tumor necrosis factor-alpha promotes in vitro calcification of vascular cells via the cAMP pathway. Circulation.

[136] Lim J., Ehsanipour A., Hsu J.J., Lu J., Pedego T., Wu A., Walthers C.M., Demer L.L., Seidlits S.K., Tintut Y. (2016). Inflammation Drives Retraction, Stiffening, and Nodule Formation via Cytoskeletal Machinery in a Three-Dimensional Culture Model of Aortic Stenosis. Am. J. Pathol..

[137] St Hilaire C., Koupenova M., Carroll S.H., Smith B.D., Ravid K. (2008). TNF-alpha upregulates the A2B adenosine receptor gene: The role of NAD(P)H oxidase 4. Biochem. Biophys. Res. Commun..

[138] De Keulenaer G.W., Alexander R.W., Ushio-Fukai M., Ishizaka N., Griendling K.K. (1998). Tumour necrosis factor alpha activates a p22phox-based NADH oxidase in vascular smooth muscle. Biochem. J..

[139] Lai C.F., Shao J.S., Behrmann A., Krchma K., Cheng S.L., Towler D.A. (2012). TNFR1-Activated Reactive Oxidative Species Signals Up-Regulate Osteogenic Msx2 Programs in Aortic Myofibroblasts. Endocrinology.

[140] Al-Aly Z., Shao J.S., Lai C.F., Huang E., Cai J., Behrmann A., Cheng S.L., Towler D.A. (2007). Aortic Msx2-Wnt calcification cascade is regulated by TNF-alpha-dependent signals in diabetic Ldlr-/- mice. Arter. Thromb. Vasc. Biol..

[141] Mitton-Fitzgerald E., Gohr C.M., Bettendorf B., Rosenthal A.K. (2016). The Role of ANK in Calcium Pyrophosphate Deposition Disease. Curr. Rheumatol. Rep..

[142] Gonzalez Rodriguez A., Schroeder M.E., Grim J.C., Walker C.J., Speckl K.F., Weiss R.M., Anseth K.S. (2021). Tumor necrosis factor-alpha promotes and exacerbates calcification in heart valve myofibroblast populations. FASEB J..

[143] McEvoy J.W., Blaha M.J., DeFilippis A.P., Lima J.A., Bluemke D.A., Hundley W.G., Min J.K., Shaw L.J., Lloyd-Jones D.M., Barr R.G. (2015). Cigarette smoking and cardiovascular events: Role of inflammation and subclinical atherosclerosis from the MultiEthnic Study of Atherosclerosis. Arter. Thromb. Vasc. Biol..

[144] McEvoy J.W., Nasir K., DeFilippis A.P., Lima J.A., Bluemke D.A., Hundley W.G., Barr R.G., Budoff M.J., Szklo M., Navas-Acien A. (2015). Relationship of cigarette smoking with inflammation and subclinical vascular disease: The Multi-Ethnic Study of Atherosclerosis. Arter. Thromb. Vasc. Biol..

[145] Choi B., Kim E.Y., Kim J.E., Oh S., Park S.O., Kim S.M., Choi H., Song J.K., Chang E.J. (2021). Evogliptin Suppresses Calcific Aortic Valve Disease by Attenuating Inflammation, Fibrosis, and Calcification. Cells.

[146] Zinman B., Wanner C., Lachin J.M., Fitchett D., Bluhmki E., Hantel S., Mattheus M., Devins T., Johansen O.E., Woerle H.J. (2015). Empagliflozin, Cardiovascular Outcomes, and Mortality in Type 2 Diabetes. N. Engl. J. Med..

[147] Alesutan I., Luong T.T.D., Schelski N., Masyout J., Hille S., Schneider M.P., Graham D., Zickler D., Verheyen N., Estepa M. (2021). Circulating uromodulin inhibits vascular calcification by interfering with pro-inflammatory cytokine signalling. Cardiovasc. Res..

[148] Xu K., Zhou T., Huang Y., Chi Q., Shi J., Zhu P., Dong N. (2018). Anthraquinone Emodin Inhibits Tumor Necrosis Factor Alpha-Induced Calcification of Human Aortic Valve Interstitial Cells via the NF-kappaB Pathway. Front. Pharm..

[149] Tintut Y., Demer L.L. (2021). Potential impact of the steroid hormone, vitamin D, on the vasculature. Am. Heart J..

[150] Price P.A., Buckley J.R., Williamson M.K. (2001). The amino bisphosphonate ibandronate prevents vitamin D toxicity and inhibits vitamin D-induced calcification of arteries, cartilage, lungs and kidneys in rats. J. Nutr..

[151] Mawer E.B., Backhouse J., Holman C.A., Lumb G.A., Stanbury S.W. (1972). The distribution and storage of vitamin D and its metabolites in human tissues. Clin. Sci..

[152] Demer L.L., Hsu J.J., Tintut Y. (2018). Steroid Hormone Vitamin D: Implications for Cardiovascular Disease. Circ. Res..

[153] Tintut Y., Abedin M., Cho J., Choe A., Lim J., Demer L.L. (2005). Regulation of RANKL-induced osteoclastic differentiation by vascular cells. J. Mol. Cell. Cardiol..

[154] Simpson C.L., Mosier J.A., Vyavahare N.R. (2021). Osteoclast-Mediated Cell Therapy as an Attempt to Treat Elastin Specific Vascular Calcification. Molecules.

[155] Simpson C.L., Lindley S., Eisenberg C., Basalyga D.M., Starcher B.C., Simionescu D.T., Vyavahare N.R. (2007). Toward cell therapy for vascular calcification: Osteoclast-mediated demineralization of calcified elastin. Cardiovasc. Pathol..

[156] Hildebrand S., Cunningham J. (2021). Is there a role for bisphosphonates in vascular calcification in chronic kidney disease?. Bone.

[157] Bartstra J.W., de Jong P.A., Kranenburg G., Wolterink J.M., Isgum I., Wijsman A., Wolf B., den Harder A.M., Mali W., Spiering W. (2020). Etidronate halts systemic arterial calcification in pseudoxanthoma elasticum. Atherosclerosis.

[158] Kranenburg G., de Jong P.A., Bartstra J.W., Lagerweij S.J., Lam M.G., Ossewaarde-van Norel J., Risseeuw S., van Leeuwen R., Imhof S.M., Verhaar H.J. (2018). Etidronate for Prevention of Ectopic Mineralization in Patients With Pseudoxanthoma Elasticum. J. Am. Coll. Cardiol..

[159] Kawahara T., Nishikawa M., Kawahara C., Inazu T., Sakai K., Suzuki G. (2013). Atorvastatin, etidronate, or both in patients at high risk for atherosclerotic aortic plaques: A randomized, controlled trial. Circulation.

[160] Marques G.L., Hayashi S., Bjallmark A., Larsson M., Riella M., Olandoski M., Lindholm B., Nascimento M.M. (2021). Osteoprotegerin is a marker of cardiovascular mortality in patients with chronic kidney disease stages 3-5. Sci. Rep..

[161] Jackson M.F., Scatena M., Giachelli C.M. (2017). Osteoclast precursors do not express CD68: Results from CD68 promoter-driven RANK transgenic mice. FEBS Lett..

[162] Rementer C.W., Wu M., Buranaphatthana W., Yang H.Y., Scatena M., Giachelli C.M. (2013). An inducible, ligand-independent receptor activator of NF-kappaB gene to control osteoclast differentiation from monocytic precursors. PLoS ONE.

[163] Nowak L.G., Rosay B., Czege D., Fonta C. (2015). Tetramisole and Levamisole Suppress Neuronal Activity Independently from Their Inhibitory Action on Tissue Non-specific Alkaline Phosphatase in Mouse Cortex. Subcell Biochem..

[164] Kiffer-Moreira T., Yadav M.C., Zhu D., Narisawa S., Sheen C., Stec B., Cosford N.D., Dahl R., Farquharson C., Hoylaerts M.F. (2013). Pharmacological inhibition of PHOSPHO1 suppresses vascular smooth muscle cell calcification. J. Bone Miner. Res..

[165] Tani T., Fujiwara M., Orimo H., Shimizu A., Narisawa S., Pinkerton A.B., Millan J.L., Tsuruoka S. (2020). Inhibition of tissue-nonspecific alkaline phosphatase protects against medial arterial calcification and improves survival probability in the CKD-MBD mouse model. J. Pathol..

[166] Barbarawi M., Kheiri B., Zayed Y., Barbarawi O., Dhillon H., Swaid B., Yelangi A., Sundus S., Bachuwa G., Alkotob M.L. (2019). Vitamin D Supplementation and Cardiovascular Disease Risks in More Than 83000 Individuals in 21 Randomized Clinical Trials: A Meta-analysis. JAMA Cardiol..

[167] Sheen C.R., Kuss P., Narisawa S., Yadav M.C., Nigro J., Wang W., Chhea T.N., Sergienko E.A., Kapoor K., Jackson M.R. (2015). Pathophysiological role of vascular smooth muscle alkaline phosphatase in medial artery calcification. J. Bone Miner. Res..

[168] Pinkerton A.B., Sergienko E., Bravo Y., Dahl R., Ma C.T., Sun Q., Jackson M.R., Cosford N.D.P., Millan J.L. (2018). Discovery of 5-((5-chloro-2-methoxyphenyl)sulfonamido)nicotinamide (SBI-425), a potent and orally bioavailable tissue-nonspecific alkaline phosphatase (TNAP) inhibitor. Bioorg. Med. Chem. Lett..

[169] Parashar A., Gourgas O., Lau K., Li J., Muiznieks L., Sharpe S., Davis E., Cerruti M., Murshed M. (2021). Elastin calcification in in vitro models and its prevention by MGP’s N-terminal peptide. J. Struct. Biol..

[170] Derwall M., Malhotra R., Lai C.S., Beppu Y., Aikawa E., Seehra J.S., Zapol W.M., Bloch K.D., Yu P.B. (2012). Inhibition of bone morphogenetic protein signaling reduces vascular calcification and atherosclerosis. Arter. Thromb. Vasc. Biol..

[171] Benitez J., Fontanarosa D., Wang J., Paritala P.K., McGahan T., Lloyd T., Li Z. (2021). Evaluating the Impact of Calcification on Plaque Vulnerability from the Aspect of Mechanical Interaction Between Blood Flow and Artery Based on MRI. Ann. Biomed. Eng..

[172] Criqui M.H., Forbang N.I., Thomas I.C. (2020). The Importance of Coronary Artery Calcium Density. JAMA Cardiol..

[173] Barrett H.E., Cunnane E.M., Kavanagh E.G., Walsh M.T. (2016). Towards the characterisation of carotid plaque tissue toughness: Linking mechanical properties to plaque composition. Acta Biomater..

[174] Herisson F., Heymann M.F., Chetiveaux M., Charrier C., Battaglia S., Pilet P., Rouillon T., Krempf M., Lemarchand P., Heymann D. (2011). Carotid and femoral atherosclerotic plaques show different morphology. Atherosclerosis.

[175] Aengevaeren V.L., Mosterd A., Braber T.L., Prakken N.H.J., Doevendans P.A., Grobbee D.E., Thompson P.D., Eijsvogels T.M.H., Velthuis B.K. (2017). Relationship Between Lifelong Exercise Volume and Coronary Atherosclerosis in Athletes. Circulation.

[176] Henein M., Granasen G., Wiklund U., Schmermund A., Guerci A., Erbel R., Raggi P. (2015). High dose and long-term statin therapy accelerate coronary artery calcification. Int. J. Cardiol..

[177] Schwartz R.S., Kraus S.M., Schwartz J.G., Wickstrom K.K., Peichel G., Garberich R.F., Lesser J.R., Oesterle S.N., Knickelbine T., Harris K.M. (2014). Increased Coronary Artery Plaque Volume Among Male Marathon Runners. Mo. Med..

[178] Chamani S., Liberale L., Mobasheri L., Montecucco F., Al-Rasadi K., Jamialahmadi T., Sahebkar A. (2021). The role of statins in the differentiation and function of bone cells. Eur. J. Clin. Investig..

[179] Hsu J.J., Fong F., Patel R., Qiao R., Lo K., Soundia A., Chang C.C., Le V., Tseng C.H., Demer L.L. Changes in microarchitecture of atherosclerotic calcification assessed by (18)F-NaF PET and CT after a progressive exercise regimen in hyperlipidemic mice. J. Nucl. Cardiol..

[180] Xian J.Z., Lu M., Fong F., Qiao R., Patel N.R., Abeydeera D., Iriana S., Demer L.L., Tintut Y. (2021). Statin Effects on Vascular Calcification: Microarchitectural Changes in Aortic Calcium Deposits in Aged Hyperlipidemic Mice. Arter. Thromb. Vasc. Biol..

